# Epigenetic mechanisms in neurological and neurodegenerative diseases

**DOI:** 10.3389/fncel.2015.00058

**Published:** 2015-02-27

**Authors:** Jorge Landgrave-Gómez, Octavio Mercado-Gómez, Rosalinda Guevara-Guzmán

**Affiliations:** Facultad de Medicina, Departamento de Fisiología, Universidad Nacional Autónoma de MéxicoMéxico, D.F., México

**Keywords:** epigenetics, neurodegeneration, DNA methylation, postranslational modification, Parkinson disease, epilepsy

## Abstract

The role of epigenetic mechanisms in the function and homeostasis of the central nervous system (CNS) and its regulation in diseases is one of the most interesting processes of contemporary neuroscience. In the last decade, a growing body of literature suggests that long-term changes in gene transcription associated with CNS’s regulation and neurological disorders are mediated via modulation of chromatin structure. “Epigenetics”, introduced for the first time by Waddington in the early 1940s, has been traditionally referred to a variety of mechanisms that allow heritable changes in gene expression even in the absence of DNA mutation. However, new definitions acknowledge that many of these mechanisms used to perpetuate epigenetic traits in dividing cells are used by neurons to control a variety of functions dependent on gene expression. Indeed, in the recent years these mechanisms have shown their importance in the maintenance of a healthy CNS. Moreover, environmental inputs that have shown effects in CNS diseases, such as nutrition, that can modulate the concentration of a variety of metabolites such as acetyl-coenzyme A (acetyl-coA), nicotinamide adenine dinucleotide (NAD^+^) and beta hydroxybutyrate (β-HB), regulates some of these epigenetic modifications, linking in a precise way environment with gene expression. This manuscript will portray what is currently understood about the role of epigenetic mechanisms in the function and homeostasis of the CNS and their participation in a variety of neurological disorders. We will discuss how the machinery that controls these modifications plays an important role in processes involved in neurological disorders such as neurogenesis and cell growth. Moreover, we will discuss how environmental inputs modulate these modifications producing metabolic and physiological alterations that could exert beneficial effects on neurological diseases. Finally, we will highlight possible future directions in the field of epigenetics and neurological disorders.

## Epigenetics

The term epigenetics is derived from the theoretical and experimental work of Conrad Waddington. He coined the term to describe a conceptual solution to a phenomenon that arises as a fundamental consideration of developmental biology (Waddington, 1942). All of the different cells in the body of one individual have exactly the same genome, that is, exactly the same DNA nucleotide sequence, with only a few exceptions in the reproductive, immune and nervous systems. Thus, in the vast majority of instances, one’s liver cells have exactly the same DNA as neurons. However, those two types of cells are clearly vastly different in terms of the gene products that they produce. Some level of mechanism must exist, Waddington reasoned, that is “above” the levels of genes encoded by the DNA sequence, which controls the DNA readout. For this reason, he defined the term *epigenetics* in the early 1940s as “the branch of biology which studies the causal interactions between genes and their products which bring the phenotype into being” (Waddington, 1968). In the original sense of this definition, epigenetics is referred to all molecular pathways modulating the expression of a genotype into a particular phenotype.

However, and with the fast expansion in this field, epigenetics has been redefined and accepted today as “the study of changes in gene function that are mitotically and/or meiotically heritable and that does not entail a change in DNA sequence.” In this way, recent advances have evolved our understanding of classical epigenetic mechanisms and the broader landscape of molecular interactions and cellular functions that are inextricably linked to these processes. The current view of epigenetics includes the dynamic nature of DNA methylation, active mechanisms for DNA demethylation, differential functions of 5-methylcytosine and its oxidized derivatives, the intricate regulatory logic of histone post-translational modifications, the incorporation of histone variants into chromatin, nucleosome occupancy and dynamics. Nevertheless, of all these modifications, the mechanisms better described in literature generally comprise histone variants, posttranslational modifications of amino acids on the amino-terminal tail of histones, and covalent modifications of DNA bases.

In this chapter, we will discuss some of these epigenetic modifications and how these modifications are associated with neurologic homeostasis and diseases.

## Linking the environment, nutrition and epigenetic modifications

**Figure 1:**
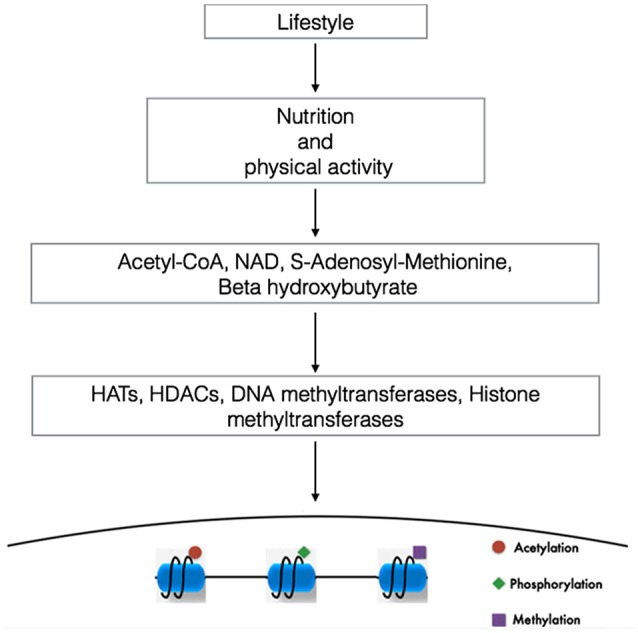
**Linking lifestyle with genome expression**. DNA and the proteins that provides chromatin structure are targets of multiple modifications. In this way, changes in our lifestyle (diets or physical activity) via the modulation of the metabolism alters the concentration ratio of different metabolites. The availability and cellular compartamentalization of these metabolites alters the activity of proteins capable to elicit epigenetic modifications, contributing to the specificity of the genome expression. NAD, nicotine adenine dinucleotide (Modified from Sassone-Corsi, 2013).

Although many aspects of nutrition and different kinds of lifestyles influence metabolic status and disease trajectory throughout our life, emerging findings suggest that changing our metabolism with exercise or different dietary regimens such as ketogenic diets, low-carbohydrate diets, intermittent fasting or physical exercise can alter the concentration of a variety of metabolites, some of them capable of modulating the activity of proteins that elicit epigenetic modifications (Figure 1; Shimazu et al., 2013; Shyh-Chang et al., 2013).

These epigenetic modifications seem to regulate important networks of genes mediating physiological processes associated with the beneficial effect of these diets, providing a rationale and simple way to prevent or even treat these diseases. Some reports have shown the efficacy of exercise and diet in cancer; cardiovascular disease, diabetes, obesity, rheumatoid arthritis and even in some neurological/neurodegenerative diseases such as Alzheimer and epilepsy (Müller et al., 2001; Ahmet et al., 2005; Belkacemi et al., 2012; Kroeger et al., 2012; Lee et al., 2012; Varady et al., 2013; Colman et al., 2014).

Consistently, some reports have shown that aging it’s a process that may be altered through some diets, such as calorie restriction (Colman et al., 2014). The precise mechanisms of how environment mediates epigenetic modifications are not clearly understood, however in this manuscript we will portray some studies that aim to epitomize the relationship between environment, metabolism, epigenetics and neurological/neurodegenerative diseases.

## Epigenetic modifications

Within cell nucleus, the fundamental units of chromatin are named nucleosomes. Each nucleosome is formed by 147 DNA base pairs wrapped tightly around an octamer of histone proteins, which is assembled by two copies of each of the four core histones (H2A, H2B, H3 and H4). The linker histone H1 binds to the DNA between the nucleosomal core particles, and their function is to stabilize higher order chromatin structures. Moreover, each histone protein consists of a central globular domain and N-terminal tail that contains multiple sites for potential modifications (Wang et al., 2013).

In this regard, a variety of different modifications on amino acid residues of histones have been described. Histone posttranslational modifications include acetylation, methylation, phosphorylation, ubiquitination and sumoylation (Table 1; Sassone-Corsi, 2013).

**Table 1:** **Histone posttranslational modifications and their role on transcription**.

Modification	Role in transcription	Modification site
**Acetylation**	Activation	H3(K9, K14, K18, K56).
		H4(K5, K8, K12, K16).
		H2B(K6, K7, K16, K17)
		(Strahl and Allis, 2000).
**Methylation**	Activation	H3(K4me2, K4me3, K36me3, K79me2)
		(Strahl and Allis, 2000)
**Methylation**	Repression	H3(K9me3, K27me3) and H4(K20me3)
		(Balazs, 2014).
**Phosphorylation**	Activation	H3(S10)
		(Strahl and Allis, 2000)

**Figure 2:**
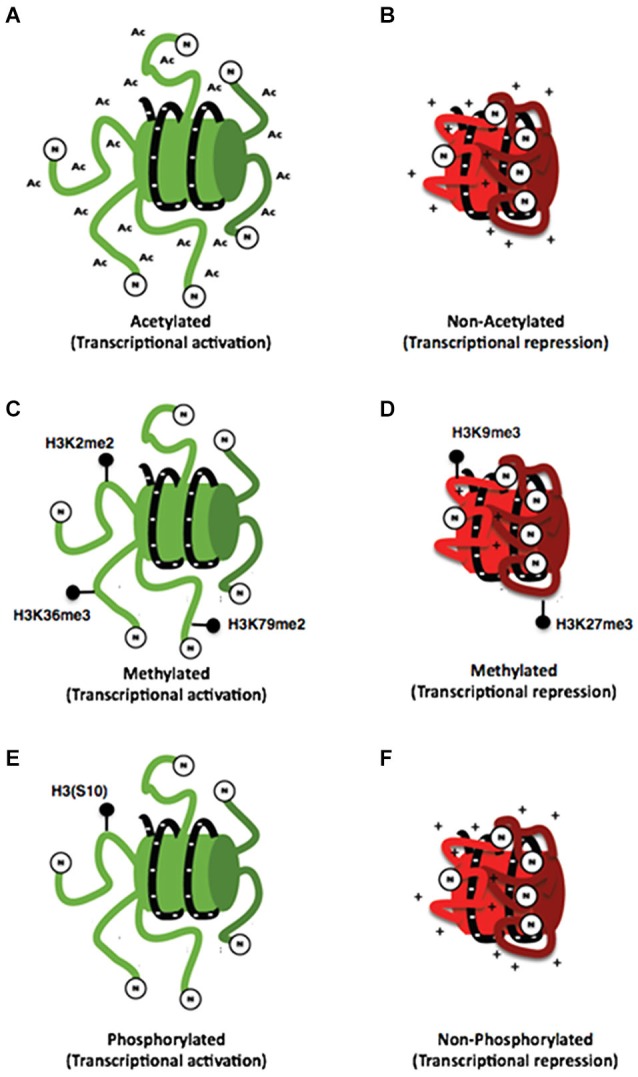
**Histone posttranslational modifications. (A)** Schemes representing the interaction of the N-terminal dominium of acetylated histones with the DNA strand **(A)** and the interaction of a non-acetylated histone with the DNA strand **(B)**. It can be noticed that acetylated histones have a minor interaction with DNA strand compared with that of non-acetylated histones whose positive charges are attracted to negative charges of DNA. **(C)** On the other hand different specific marks of methylation of histone 3 are associated with both transcriptional activation **(C)** and repression **(D)**. Also a specific mark of phosphorylation on the (S10) amino acid of histone 3 has been associated with transcriptional activation **(E)** so the lack of this mark may be associated with transcriptional repression **(F)**.

The principal residues that are substrates of these modifications are lysine, arginine, serine and threonine amino acids (Rothbart and Strahl, 2014). These modifications have been associated to repression or activation of gene transcription depending on the site of the modification, strongly suggesting the existence of a histone code. This hypothesis proposes that specific modifications of histones induce to the interaction with proteins associated with the chromatin, producing a differential regulatory response of gene expression (Strahl and Allis, 2000; Table 1 and Figure 2). These modifications are dynamic in the way that they are actively added and removed by histone-modifying enzymes in a site-specific manner, which is essential for coordinated transcriptional control.

## Histone acetylation

The acetylation of histones is a modification associated generally to transcriptional activity that indicates access of the transcription machinery to the genes and thus active mechanisms (Strahl and Allis, 2000; Balazs, 2014). This effect could be explained by the chemistry of this modification in which an acetyl group (−COCH3) is incorporated to an amino terminal residue and thus, the positive charge of histones is reduced, inducing a minor interaction with DNA resulting in a decrease of the chromatin compaction (Figures 2A,B; Shahbazian and Grunstein, 2007).

## Histone methylation

Histone methylation is currently associated with multiple processes such as transcriptional activation and repression, depending on the modified amino acid residue (Figures 2C,D). This modification occurs mainly on arginine and lysine residues. Additionally, these residues could be methylated multiple times giving different signals depending on how many times the residue is methylated, making its analysis difficult. In this regard, current literature has shown that lysine residues can be methylated even three times; meanwhile, arginine residues can only be methylated twice (Strahl and Allis, 2000). Furthermore, there have been some studies associating some processes with these types of modifications for example H3K4, H3K36 and H3K79 are associated with chromatin aperture. Nevertheless, the methylation of these residues has been also associated with other specific functions. On the other hand, H3K4 trimethylation has been associated with promoter regions. The monomethylathion of this same residue recruits regulatory elements that potentiate the promoter activity; such elements are known as *enhancers*. Dimethylation of H3K36 has been related to RNA POL II elongation during transcription (Li et al., 2007). Also, the dimethylation of H3K79 is particular of promoter regions stimulating a permissive chromatin for local transcription (Jacinto et al., 2009). Conversely, the modifications associated with transcriptional repression are performed on H3K9 and H3K27 residues (Baylin and Jones, 2011).

## DNA methylation

**Figure 3:**
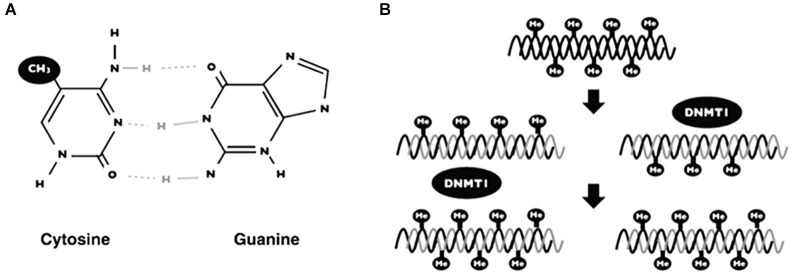
**DNA methylation**. Scheme showing the addition of a methyl group on the 5 carbon of cytosine in the context of 5′-CpG-3′ dinucleotide **(A)**. The maintenance of DNA methylation is accomplished by DNA methyl-transferases (DNMT1) when DNA replication occurs **(B)**.

In mammalians, DNA methylation is the covalent union of methyl groups of cytosines that are found mainly in the context of dinucleotide 5′-CpG-3′ (Figure 3A; Klose and Bird, 2006). The addition of methyl groups protrudes above the major groove and when DNA is symmetrically methylated, the methyl groups promote a conformational change of DNA structure. The main consequence of methyl modification is that a variety of transcription factors cannot recognize the DNA and thus induce repressional transcription (Prokhortchouk and Defossez, 2008).

DNA methylation generates patterns that are established during embryonic development and such patterns are maintained by a mechanism when DNA replicates (Figure 3). Interestingly, these patterns change over time, principally due to environmental factors (i.e., nutrition, metabolites, exercise, chemical agents) (Fraga et al., 2005). The mechanism of DNA methylation is carried out by a set of proteins named DNA methyl-transferases (DNMTs). There are two groups of these proteins; (1) one for *de novo* methylation; and (2), one for methylation maintenance. Both enzymes differ depending on the DNA substrate: for example, maintenance of DNA methylation is accomplished by DNA methyl transferase 1 (DNMT1). These proteins add methyl groups to pre-existing methyl patterns on a new strand of DNA during replication (Figure 3B; Jeltsch, 2006). On the other hand, *de novo* DNA methylation are carried out by DNMT3a and DNMT3b. Such proteins are responsible for the addition of new methyl groups to cytosines that have not been methylated previously (Goll and Bestor, 2005).

## Histone variants

Histone variants such as H2A and H3.3 have been known since several decades ago and recently, a lot of evidence has been accumulated about their role in their participation on the differential structure of chromatin (Henikoff et al., 2004). Among them, H2A.Z has been located on DNA regions associated with transcriptional activation, mainly, on promoter regions. This variant is important because it induces a less stable structure of chromatin compared with that of the canonical histone H2 (Draker and Cheung, 2009). Another histone variant associated with promoter regions is H3.3. This variant as well as H2A.Z, is mainly found on promoter regions suggesting that their structure promotes the formation of a more permissive chromatin (Jin et al., 2009).

## Neuroepigenetics and their role in neuronal function

Over the last two decades, the field of epigenetics, particularly the emerging field of neuroepigenetics, has begun to have a great impact in different areas such as the study of the CNS development, learned behavior, neurotoxicology, cognition, addiction and lately neurological and neurodegenerative pathology (Sweatt, 2013). In this regard, epigenetics has undergone an exponential expansion. A quick search of the PubMed database reveals that about 98% of all the research work on epigenetics was published within the last 15 years (Sweatt, 2013). Thanks to these studies, nowadays we know that either maternal behavior, environmental toxins, nutrition, physociological or physical stress, learning, drug exposure or psychotrauma, leads to active regulation of the chemical and three-dimensional structure of DNA and thus, regulates epigenetics modifications in the CNS linking environmental *stimuli* and gene expression regulation (Tsankova et al., 2007; Borrelli et al., 2008; Renthal and Nestler, 2008; Champagne and Curley, 2009; Day and Sweatt, 2010; Dulac, 2010).

These epigenenomic changes allow perpetual alterations in gene readout in cells in the CNS affecting neuronal function and physiology. For example, a central regulator of homeostasis in the brain, the brain-derived neurotrophic factor (BDNF), a member of the neurotrophin family of proteins that plays crucial roles in the development, maintenance, and plasticity of the CNS (Chao et al., 2006) have been demonstrated to play an important role on different psychiatric disorders associated with early-life adversity, including depression; schizophrenia, bipolar disorder and autism. Even when the underlying mechanisms of the alterations over the expression of BDNF are unknown in these conditions, epigenetic modifications seem as a plausible candidate, as early-life exposures, chronic emotional *stimuli*, or even emotional behavior, disrupts epigenetic programming in the brain with lasting consequences for gene expression and behavior (Renthal et al., 2007; LaPlant et al., 2010; Kundakovic et al., 2014).

However, epigenetics is such a new field of science that in most of the cases, its impact has not been fully demonstrated. Even though, it is now clear that there is a dynamic interplay between genes and experience, a clearly delineated and biochemically driven mechanistic interface between genes and environment, this interface is epigenetics (Sweatt, 2013).

## Alzheimer’s disease and epigenetics

Alzheimer’s disease (AD) is an age-related and slowly neurodegenerative disorder of the brain and the most common form of dementia in the elderly (Sezgin and Dincer, 2014). The disease is clinically characterized by progressive memory loss and cognitive impairment. Moreover, the histopathological features of AD are senile plaques composed of amyloid beta (Aβ) fibrils and neurofibrillary tangles composed of microtubule-associated protein tau, combined with massive cholinergic neuronal loss, mainly in the hippocampus and association regions of neocortex (Hardy, 2006; Ballatore et al., 2007). This disease currently affects approximately 2% of the population in industrialized countries and its incidence will increase dramatically over the time (Sezgin and Dincer, 2014).

AD is a multifactorial disease involving; genetic, metabolic, nutritional, environmental and social factors that are associated with onset and progression of the pathology. For this reason, and considering that the main risk factor of this disorder is aging, it is reasonable to think that life history such hypertension, diabetes, inflammation, obesity or head injury are closely related with AD (Marques et al., 2011). However, how these factors induce epigenetic changes that mediate the network genes involved in this disease is a question that remains to be answered.

At present, studies of epigenetic changes in AD are starting to emerge. As we mentioned before, aging is the most important risk factor for AD an epigenetic changes have been observed in aging tissues. Recently, it has been observed that environmental factors even transient ones in early life can induce AD-like pathogenesis in association with aging (Wu et al., 2008a). Furthermore, a difference in DNA methylation patterns typical of brain region and aging has been identified in this context (Balazs, 2014). In this regard, a recent study by Hernandez et al. examined the DNA methylation patterns in >27,000 CpG sites from donors ranging in age 4 months to 102 years and a strong relationship was found between DNA methylation and aging. Moreover, in the temporal and frontal cortices pons and cerebellum regions, more than 1,000 associations were found between DNA methylation at CpG sites and age and some associations were significant in all four regions. Interestingly, the majority of the association sites were in CpG islands and the pattern was similar in the frontal cortex, temporal cortex and pons, but different in cerebellum. These results suggest that and age-dependent increase in DNA methylation may be important for maintaining gene expression with age (Hernandez et al., 2011).

As it has been reported in many studies, memory can be compromised during aging. Preclinical and basic studies have shown that epigenetic mechanisms are involved in formation and maintenance of memory (for reviews, see Levenson and Sweatt, 2005; Zovkic et al., 2013; Jarome et al., 2014). For example, inhibition of DNA methylation has deleterious effects on neuronal plasticity together with histone modifications (Day and Sweatt, 2011; Zovkic et al., 2013). Moreover, it has been observed that associative learning was impaired in 16-month-old mice compared with that of 3-month-old mice which was associated with specific reduction in acetylation of H4K12 (Peleg et al., 2010).

Until now, most of the studies have analyzed DNA methylation in the brain of AD patients (Balazs, 2014). In this regard, a variety of studies suggest a genome-wide decrease in DNA methylation present in aging and AD patients (Table 2; Mastroeni et al., 2011). Interestingly, the folate/methionine metabolism is critically linked with DNA methylation mechanisms, consistently with this fact; studies show that folate and S-adenosyl methionine are significantly decreased in AD (Bottiglieri et al., 1990; Morrison et al., 1996). All this data indicates that AD patients produce a hypomethylation across the DNA genome. Recently, Bakulski et al. provided a semi-unbiased, quantitative, genome-wide localization of DNA epigenetic differences in frontal cortex of control and AD cases. These authors determined DNA methylation of 27, 587 CpG sites spanning 14,475 genes. Interestingly, they found that in control samples, the methylation state is markedly affected by age, with about the same number of sites being hypermethylated as hypomethylated with age. Compared with controls, 6% of genes featured on the array were differentially methylated in AD samples, but the mean difference was relatively modest (2.9%). Gene ontology analysis revealed a relationship between the main disease-specific methylation loci and several molecular functions and biological processes, including hypermethylation of genes involved in transcription and DNA replication, while membrane transporters were hypomethylated (Bakulski et al., 2012).

**Table 2:** **Epigenetic modifications implicated in Alzheimer’s disease**.

Observation	Sample
APP promoter hypomethylation in Alzheimer’s disease patients (Miller, 2003).	Human brains.
Hypomethylation of promoters of ribosomal genes with aging (Decottignies and d’Adda di Fagagna, 2011).	Human lymphocytes.
Decrements in DNA methylation (Al-Mahdawi et al., 2014).	Human prefrontal cortex.
Differences in DNA methylation in a twin pair discordant for Alzheimer’s disease (Al-Mahdawi et al., 2014).	Human temporal neocortex.
APP promoter methylation influenced by sex steroids and aging (Maloney et al., 2012).	Intact and gonadectomized mice brains.
PSEN1 is regulated by DNA methylation in response to metabolic stimuli (Zetzsche et al., 2010).	Non-human primate cortical areas of mice brains.

Also, some reports have focused on research DNA methylation at the 5′ promoter regions of candidate genes according to the basis of hypothesis concerning the molecular mechanisms of AD as microtubule-associated protein tau, amyloid precursor protein (APP) and preseniline-1 genes in the frontal cortex and hippocampus of both control and AD cases at different Braak stages. Interestingly, there wasn’t any significant difference on CpG methylation between the control and AD samples (Barrachina and Ferrer, 2009). Other studies have reported hypomethylation of APP in the promoter region of normal 70 year-old human brain (Tohgi et al., 1999). However, as mentioned above, no difference was found in methylation of selected regions of the APP gene in various stages of AD progression (Barrachina and Ferrer, 2009). Also, it has been found that the change in methylation status differed among transcription factor binding sites of tau promoter (Wang et al., 2013).

Additionally to DNA methylation, histone modifications have been studied in recent years. Francis et al. investigated histone acetylation in mouse models of AD. In APP/presenilin1 double mutant transgenic mice, associative learning was impaired and this was linked to a marked reduction in H4K14 histone acetylation (Francis et al., 2009). Furthermore, studies *in vitro* have shown that exposure of cortical and hippocampal cultures to Aβ oligomers resulted in increased levels of acetylated H3K14 and a loss of dendritic spines, which was prevented by inhibition of histone acetyl transferase. Also, in young pre-plaque AD transgenic mice, these authors observed markedly increased levels of H3K14 and H3K9me2 compared with those of wild-type non-transgenic mice. Most importantly, similar changes were observed in histone transcription activating and repressive marks in the occipital cortex of AD samples (Lithner et al., 2013).

Although there are now treatments against AD, these are only palliatives and the pathology is currently incurable, whereby, there is an intense interest in the development of new potential therapies. Epigenetic therapies have achieved some progress in the field of cancer, thus, several inhibitors of HDACs and DNA methylation are approved for hematological malignances by the US Food and Drug Administration and have been in clinical use for several years (Wu et al., 2008a). HDAC inhibitors (HDACIs) are the most thoroughly studied and have shown acceptable results in AD models. The inhibitors widely used in clinical research include trichostatin A (TSA), valproic acid (VPA), sodium 4-phenylbutyrate (4-PBA) and vorinostat (SAHA) (Wang et al., 2013).

In a study conducted by Su et al., VPA showed to inhibit Aβ production in HEK293 cell transfected with a plasmid carrying the Swedish APP751 mutation. Interestingly, using the APPV717F transgenic model of AD, VPA was able to inhibit Aβ production in the brain of mice at biologically relevant doses of 400 mg/kg (Su et al., 2004). In another study, VPA showed to decrease Aβ production and alleviate behavioral deficits by inhibiting GSK-3β-mediated γ-secretase cleavage of APP in APP23 transgenic mice (Qing et al., 2008). These results give us the idea about the possible contribution of epigenetic modifications in AD, which suggests that the drugs targeting epigenetic process may be of future therapeutic value (Wang et al., 2013).

As mentioned widely in scientific literature, the interaction between diet and epigenetics is the best documented in cancer pathology (Ho et al., 2009; Shu et al., 2010). Furthermore, based on evidence in support of epigenomics in regulating gene expression in stress-mediated AD risk factors, and the pathophysiology of AD, there has been growing interest in examining whether diet and nutraceuticals targeting epigenomics may prevent, delay, or reverse the course of AD (Chiu et al., 2014). In this regard, the Mediterranean diet rich in vegetables, fruits and nuts, legumes, olive oil and fish with relative low intakes of red meat has been suggested to reduce the risk for AD onset (Scarmeas et al., 2009; Frisardi et al., 2010). Other studies appoint that anti-oxidant-rich diets and consumption of dietary phytochemical such as caffeic acid, epigallocatechin-3-gallate, *Gingko biloba*, resveratrol and phenolic compounds present in red wine slowed down disease progression by inhibiting Aβ production or amyloid aggregation in animal models (Kolosova et al., 2006).

It is well known that DNA methylation occurs within folate/methionine/homocysteine (HCY) metabolism which uses micronutrients such as folate, methionine, choline and betaine enzyme’s cofactors (Chouliaras et al., 2010; Wang et al., 2013). Diverse reactions occur and methionine is converted to S-adenosyl-methionine (SAM) and then converted to S-adenosyl-homocysteine (SAH), which in turn is converted to HYC in a reversible reaction. Most important, SAM is the common methyl donor for DNA methylation that regulates gene expression and determines the chromosome conformation (Sezgin and Dincer, 2014). An early study showed that SAM levels have been found to be decreased in post-mortem AD patients (Morrison et al., 1996). Also, lower bioavailability of SAM causes changes in the expression of genes involved in APP metabolism because this metabolite maintains the appropriate methylation of genes involved in APP processing (Sezgin and Dincer, 2014). Fuso et al. recently reported that reduction of folate and Vitamin B12 in culture medium of neuroblastoma cell lines cause a reduction in SAM levels resulting in an increase of PSEN1 and BACE levels together with Aβ production. Conversely, the simultaneous administration of SAM to the deficient medium restored the normal gene expression and reduced the Aβ levels (Fuso et al., 2007). Interestingly, the same group demonstrated that Vitamin B deficient-animals have shown that SAM inhibits the increase in progression of Alzheimer-like features (Fuso et al., 2012). This data suggests that folate or Vitamin B12-rich diets could be beneficial as therapy for AD patients; however, more studies are needed.

## Parkinson’s disease and epigenetics

Parkinson’s disease (PD) is the second most common neurodegenerative disorder after AD affecting approximately 1–2% of the population over the age of 65 and reaching a prevalence of almost 4% in those aged above 85. Resting tremor, bradykinesia, rigidity, and postural instability are the main clinical symptoms of the disease often accompanied by non-motor symptoms including autonomic insufficiency, cognitive impairment, and sleep disorders (Thomas and Beal, 2011; Coppedè, 2014). The brain of PD individuals is pathologically characterized by a progressive loss of neuromelanin containing dopaminergic neurons in the *substantia nigra* with the presence of eosinophilic, intracytoplasmic inclusions termed as Lewy bodies (structures containing aggregates of α-synuclein as well as other substances) and Lewy neurites in surviving neurons. Unfortunately, only some improvements of the symptoms are offered by current treatments based on levodopa and dopaminergic therapy, but there is no currently available treatment to avoid the progression of the disease (Thomas and Beal, 2011; Coppedè, 2014).

The vast majority of PD cases are idiopathic forms, likely resulting from a combination of polygenic inheritance, environmental exposures, and complex gene-environment interactions imposed on slow and sustained neuronal dysfunction due to aging (Migliore and Coppedè, 2009). In a minority of the cases, PD is inherited as Mendelian trail, and studies in PD families allowed the identification of at least 15 PD loci (PARK1-15) and several causative genes (Nuytemans et al., 2010). In addition, there are genes such as LRRK2, SNCA, MAPT and GBA that are associated with sporadic PD without family history (Table 3; Coppedè, 2012).

**Table 3:** **Epigenetic modifications of Parkinson’s disease related genes**.

Gene	Observation
***SNCA***	Reduced SNCA methylation in the substantia nigra of PD patients.
	SNCA gene silencing mediated by histone methylation (Nalls et al., 2014).
	Histone deacetylases inhibitors are neuroprotective against ***α***-synuclein mediated neurotoxicity in PD animal models (IPDGC, 2011).
***LRRK2***	*Mutant LRKK2 antagonizes miR-184 in Drosophila melanogaster Parkinson’s disease models* (IPDGC, 2011).
***Parkin***	*let-7 family miRNAs were under-expressed in parkin transgenic C.elegans* (Asikainen et al., 2010)
***PARK16/lq32, GPNMB***	*Aberrant gene methylation in post-mortem Parkinson’s disease brains* (IPDGC, 2011)

Most of the studies evaluating the role of epigenetic in pathogenesis have focused on the analysis of promoter methylation of causative PD genes in post-mortem brains and peripheral blood; however, the role of DNA methylation and its links to PD pathogenesis is currently unclear (Coppedè, 2012). Recent studies have shown that methylation of SNCA gene (the gene coding for α-synuclein) may be involved in disease via structural changes or overexpression of the protein, leading to protein aggregation or via impaired gene expression (Ammal Kaidery et al., 2013). In this regard, methylation of SNCA intron 1 has been demonstrated to be associated with decreased SNCA transcription, whereas reduced methylation at this site was found to be decreased in several brain regions, including the *subtancia nigra* of sporadic patients, causing the increased expression of the SNCA gene (Jowaed et al., 2010). These results raise the possibility that the increased α-synuclein production that is associated with PD may result from increased SNCA expression, as a consequence of a decreased methylation state of its gene (Ammal Kaidery et al., 2013). Additionally, it has been demonstrated that α-synuclein sequesters DNMT1 in the cytoplasm, leading to global DNA hypomethylation in PD and dementia with Lewy body in post-mortem brains, as well as in transgenic mouse models (Desplats et al., 2011). Conversely, the overexpression DNMT1 in both transgenic mouse models and cellular cultures restore the nuclear level of the enzyme (Ammal Kaidery et al., 2013).

The regulation of SNCA by epigenetic histone modifications is yet to be studied in human PD brains. Studies in cell cultures and animal models of the disease, such as those induced by mitochondrial toxins, including 1-methyl-4-phenylpyridinium (MPP^+^), paraquat, rotenone, or those overexpressing human α-synuclein, have revealed that α-synuclein translocates into the nucleus interacting with histones and inhibiting histone acetylation (Goers et al., 2003). Furthermore, in Drosophila models, nuclear-targeted α-synuclein has been shown to bind to histones and reduce histone 3 acetylation through its association with HDAC1 and SIRT2 (Kontopoulos et al., 2006).

In recent years, there has been considerable progress in the development of epigenetic-based drugs for the treatment of neurodegenerative disorders such as PD. Such inhibitors of HDACs and DNMTs are currently approved and available for clinical investigation (Xu et al., 2012). In this regard, the targeted downregulation of SIRT2 has been shown to ameliorate α-synuclein toxicity and dopaminergic loss in flies and in primary mesencephalic culture. Moreover, toxicity associated with nuclear-targeted α-synuclein in both SH-SY5Y neuroblastoma cells and flies can be rescued by using HDACIs (Outeiro et al., 2007), thus, HDACIs have been theorized to be efficacious in neurodegenerative diseases (Harrison and Dexter, 2013). In this regard, Wu et al. demonstrated that trichostain A (a well-known HDAC inhibitor), protects dopaminergic neurons from MPP^+^ toxicity in primary neuron-glia co-cultures in a dose dependent manner (Wu et al., 2008b). Moreover, Kid and Schneider demonstrated that vorinostat (another HDAC inhibitor) protected two different dopaminergic neuronal cell lines from apoptosis induced by MPP^+^ (Kidd and Schneider, 2010), thus, the above results give us an idea about the alternative therapy by inhibiting HDACs in PD patients.

Although the etiology of PD is still unknown, multiple lines of evidence support oxidative stress and mitochondrial dysfunction as part of the pathogenic cascade. It would be interesting to know whether antioxidants-rich diets that have a helpful effect in other degenerative disease such as AD (Kolosova et al., 2006), could have the same effect in PD patients. To this regard, therapy focusing on nutrition, neutraceutical and antioxidants as part of a healthy lifestyle might protect against cell death and thus delay or halt disease progress; however, clinical and basic studies are needed to prove such hypothesis (Bega et al., 2014).

## Epilepsy and epigenetics

Epilepsy is the third most common chronic brain disorder affecting 50 million of people worldwide (Aroniadou-Anderjaska et al., 2008). In this disorder, a variety of structures of the central nervous system such as the hippocampus, the amygdala and the piriform cortex are susceptible to trigger electrical discharges that contribute to brain damage and to the epileptogenic mechanism (Houser, 1990; Blümcke et al., 1999). These discharges promote some morphological changes in the hippocampus such as, cellular death in the CA1 and mossy fiber sprouting and dispersion of the granule cell layer, alterations that are thought to be involved in the formation of recurrent excitatory circuits that contributes to seizure susceptibility (Heck et al., 2004).

In this regard, it is well known that seizures can give rise to enduring changes that reflect alterations in gene expression patterns, contributing in this way to the hallmarks of epilepsy (Roopra et al., 2012). Moreover, some studies suggest that these long-term changes mediated by seizures are mediated via modulation of chromatin structure. One transcription factor in particular, the repressor element 1-silencing transcription factor (REST/NRSF) has received a lot of attention due to its association with a great sub-set of genes associated with important processes involved in neuronal homeostasis and because it may seem to recruit a variety of proteins that elicit epigenetic modifications such as histone deacetylases and histone methyltransferases (Bruce et al., 2004; Ballas and Mandel, 2005; Ballas et al., 2005; Johnson et al., 2006; Pozzi et al., 2013). Some reports have shown that the induction of seizures in animal models induce an overexpression in both REST/NRSF protein and mRNA levels (Formisano et al., 2007; Noh et al., 2012), suggesting that seizures may cause an unbalance in the epigenetic modifications that control important processes of neuronal homeostasis. In contrast, recent studies have shown that REST/NRSF is induced in the aging human brain regulating a network of genes associated with stress resistance (Lu et al., 2014). This evidence suggests that REST/NRSF regulates important processes in embryonic and adult neuronal homeostasis and that the dysregulation of this transcription factor may impair epigenetic modifications that regulate precisely an important network of genes contributing to distinct neurological/ neurodegenerative disorders such as epilepsy or AD.

From a public health perspective, an alternative for the treatment of epilepsy is a change of lifestyle or diet. These methods have probably been used for over 2000 years and actually metabolic regulation of neuronal excitability is increasingly recognized as a factor in seizure pathologies and control (Stafstrom et al., 2008; Yuen and Sander, 2014). In this way, approximately half of the pharmacoresistant patients that have tried metabolism based therapies experience seizure control, opening the possibility of a strong link between the environments, in this case nutrition, with this pathology (Greene et al., 2003; Bough et al., 2006; Marsh et al., 2006; Patel et al., 2010).

These studies suggest that metabolism-based therapies such as ketogenic diets, calorie restriction or intermittent fasting leads to a range of biochemical and metabolic changes that induce a metabolic shift in pathways such as glycolysis, ketogenesis or beta oxidation, modifications that have been shown to increase seizure thresholds and to decrease epileptogenesis in animal models (Marsh et al., 2006; Patel et al., 2010).

Moreover, recent studies have shown that environmental inputs such as nutrition or exercise modulates cell metabolism, and critical links between metabolism and epigenetic control are beginning to emerge (Sassone-Corsi, 2013). For example, the availability of specific metabolites such as acetyl-coenzyme A (acetyl-coA) and nicotinamide adenine dinucleotide (NAD^+^) dictates the efficacy of histone deacetylases (Katada et al., 2012).

In this regard, it has been shown that beta hydroxybutyrate (β-HB), a ketone body that rises with ketogenic diets, during strenuous exercise or during fasting (Newman and Verdin, 2014), acts as an endogenous inhibitor of histone deacetylases linking in a precise way metabolism, epigenetics and epilepsy (Shimazu et al., 2013). Thus, these studies strongly suggest that the neuroprotective effects exerted by these kinds of therapies are not only mediated via metabolism alterations but also by epigenetic modifications that may be involved in the expression of an unknown sub-set of genes related to epilepsy.

Other interesting epigenetic modifications involved in epilepsy are methylation of DNA. In this field, Kobow et al. using Methyl-seq, mapped for the first time the global DNA methylation patterns in chronic epileptic rats; they showed that chronic epilepsy in animal models is characterized for a global hypermethylation on DNA. Moreover, this group shows that ketogenic diets diminish this increase of DNA methylation, suggesting that these kinds of therapies exert their effect not only modulating metabolism, but also via epigenetic modifications (Kobow et al., 2013). More importantly, it opened the possibility for the development of new metabolism based therapies designed to regulate these epigenetic modifications contributing to the inhibition of the seizure threshold in epilepsy.

## The role of REST/NRSF in neurological disorders

**Figure 4:**
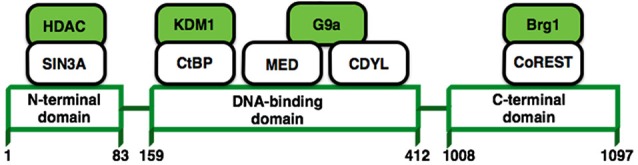
**REST structure and their interactions with other proteins**. REST modulates the expression of its target genes by recruiting a host of lysine-modifying enzymes. Numbers refer to amino acid residues. Colored molecules possess enzymatic activity.

A growing body of literature suggests that long-term changes in gene transcription associated with a lot of neurological disorders are mediated via modulation of chromatin structure. One transcription factor in particular, REST/NRSF (repressor element 1-silencing transcription factor) (Figure 4), has received a lot of attention due to the possibility that it may control the expression of approximately 1,300 genes (Bruce et al., 2004; Johnson et al., 2006) that could be associated with a variety of processes that are important for neuronal homeostasis such as; synaptic transmission, synaptogenesis, excitability or even neurogenesis (Ballas and Mandel, 2005; D’Alessandro et al., 2009). REST modulates these genes in the nervous system recruiting protein complexes that elicit different epigenetic modifications (Figure 4; Roopra et al., 2012). Now it has been shown that REST is upregulated in pyramidal and dentate gyrus neurons after *status epilepticus* induced by kainate (Palm et al., 1998) or even by ischaemic insults (Formisano et al., 2007; Noh et al., 2012). Therefore, the upregulation of REST has been previously considered as harmful in mature neurons. In contrast, recent studies have shown that induced expression of REST/NRSF in mature hippocampal neurons is a protective mechanism that modulates the inhibitory homeostatic control of intrinsic excitability (Pozzi et al., 2013). Moreover, it has been shown that REST/NRSF protects neurons from age-related toxic insults in AD and surprisingly these levels seems to be associated with preservation of cognitive function and increased longevity (Lu et al., 2014). These findings suggest that basal levels of REST/NRSF are necessary for a normal physiological condition in the adult brain and that elevated levels of REST/NRSF, characteristic of epilepsy, may not be an epileptogenic factor, rather it seems to be a homeostatic mechanism triggered by repeated hyper-excitability *stimuli*. This is an open issue that needs further investigation.

## Concluding remarks

As we state in this manuscript, one of the main factors that contributes to a variety of the most common diseases is the environment. Many epigenetic enzymes are potentially susceptible to changes in the levels of a variety of metabolites, and are, hence, poised to respond to changes on environment. In this sense, it has been demonstrated that changing our lifestyle could mediate great beneficial effects regulating a network of genes via the modulation of chromatin structure, providing new alternatives for the prevention of many diseases.

Different questions remain to be answered including which epigenetic modifications are implicated in neurological disorders, how does the environment mediate these changes, could pharmacological inhibitors of these modifications provide an alternative for treating disease, and so on. Increasing evidence on this field had taught us that these modifications are capable of regulating great networks of genes that can influence a variety of physiological processes important for overall homeostasis and that the disruption of this balance can increase the risk of disease.

From a public health perspective, we need to better understand which alterations in metabolism and in chromatin structure cause disease and, maybe, it will be possible to design rationale metabolism-based therapies that could function as alternative treatments of these kinds of disorders.

## Conflict of interest statement

The authors declare that the research was conducted in the absence of any commercial or financial relationships that could be construed as a potential conflict of interest.

## References

[B1] Ahmet I. Wan R. Mattson M. P. Lakatta E. G. Talan M. (2005). Cardioprotection by intermittent fasting in rats. Circulation 112, 3115–3121. 10.1161/CIRCULATIONAHA.105.563817 16275865

[B2] Al-Mahdawi S. Virmouni S. A. Pook M. A. (2014). The emerging role of 5-hydroxymethylcytosine in neurodegenerative diseases. Front. Neurosci. 8:397. 10.3389/fnins.2014.00397 25538551 PMC4256999

[B3] Ammal Kaidery N. Tarannum S. Thomas B. (2013). Epigenetic landscape of Parkinson’s disease: emerging role in disease mechanisms and therapeutic modalities. Neurotherapeutics 10, 698–708. 10.1007/s13311-013-0211-8 24030213 PMC3805874

[B4] Aroniadou-Anderjaska V. Fritsch B. Qashu F. Braga M. F. (2008). Pathology and pathophysiology of the amygdala in epileptogenesis and epilepsy. Epilepsy Res. 78, 102–116. 10.1016/j.eplepsyres.2007.11.011 18226499 PMC2272535

[B5] Asikainen S. Rudgalvyte M. Heikkinen L. Louhiranta K. Lakso M. Wong G.. (2010). Global microRNA expression profiling of caenorhabditis elegans Parkinson’s disease models. J. Mol. Neurosci. 41, 210–218. 10.1007/s12031-009-9325-1 20091141

[B6] Bakulski K. M. Dolinoy D. C. Sartor M. A. Paulson H. L. Konen J. R. Lieberman A. P.. (2012). Genome-wide DNA methylation differences between late-onset alzheimer’s disease and cognitively normal controls in human frontal cortex. J. Alzheimers Dis. 29, 571–588. 10.3233/JAD-2012-111223 22451312 PMC3652332

[B7] Balazs R. (2014). Epigenetic mechanisms in Alzheimer’s disease. Degener. Neurol. Neuromuscul. Dis. 4, 85–102 10.2147/dnnd.s37341 PMC7337154 32669903

[B8] Ballas N. Grunseich C. Lu D. D. Speh J. C. Mandel G. (2005). REST and its corepressors mediate plasticity of neuronal gene chromatin throughout neurogenesis. Cell 20, 645–657. 10.1016/j.cell.2005.03.013 15907476

[B9] Ballas N. Mandel G. (2005). The many faces of REST oversee epigenetic programming of neuronal genes. Curr. Opin. Neurobiol. 15, 500–506. 10.1016/j.conb.2005.08.015 16150588

[B10] Ballatore C. Lee V. M. Trojanowski J. Q. (2007). Tau-mediated neurodegeneration in Alzheimer’s disease and related disorders. Nat. Rev. Neurosci. 8, 663–672. 10.1038/nrn2194 17684513

[B11] Barrachina M. Ferrer I. (2009). DNA methylation of Alzheimer disease and tauopathy-related genes in postmortem brain. J. Neuropathol. Exp. Neurol. 68, 880–891. 10.1097/nen.0b013e3181af2e46 19606065

[B12] Baylin S. B. Jones P. A. (2011). A decade of exploring the cancer epigenome-biological and translational implications. Nat. Rev. Cancer 11, 726–734. 10.1038/nrc3130 21941284 PMC3307543

[B13] Bega D. Gonzalez-Latapi P. Zadikoff C. Simuni T. (2014). A review of the clinical evidence for complementary and alternative therapies in Parkinson’s disease. Curr. Treat. Options Neurol. 16:314. 10.1007/s11940-014-0314-5 25143234

[B14] Belkacemi L. Selselet-Attou G. Hupkens E. Nguidjoe E. Louchami K. Sener A.. (2012). Intermittent fasting modulation of the diabetic syndrome in streptozotocin-injected rats. Int. J. Endocrinol. 2012:962012. 10.1155/2012/962012 22291702 PMC3265126

[B15] Blümcke I. Beck H. Lie A. A. Wiestler O. D. (1999). Molecular neuropathology of human mesial temporal lobe epilepsy. Epilepsy Res. 36, 205–223. 10.1016/s0920-1211(99)00052-2 10515166

[B16] Borrelli E. Nestler E. J. Allis C. D. Sassone-Corsi P. (2008). Decoding the epigenetic language of neuronal plasticity. Neuron 60, 961–974. 10.1016/j.neuron.2008.10.012 19109904 PMC2737473

[B17] Bottiglieri T. Godfrey P. Flynn T. Carney M. W. Toone B. K. Reynold E. H. (1990). Cerebrospinal fluid S-adenosyl-methionine in depression and dementia: effects of treatment with parenteral and oral S-adenosyl-methionine. J. Neurol. Neurosurg. Psychiatry 53, 1096–1098. 10.1136/jnnp.53.12.1096 2292704 PMC488323

[B18] Bough K. J. Wetherington J. Hassel B. Pare J. F. Gawryluk J. W. Greene J. G.. (2006). Mitochondrial biogenesis in the anticonvulsant mechanism of the ketogenic diet. Ann. Neurol. 60, 223–235. 10.1002/ana.20899 16807920

[B19] Bruce A. W. Donaldson I. J. Wood I. C. Yerbury S. A. Sadowski M. I. Chapman M.. (2004). Genome-wide analysis of repressor element 1 silencing transcription factor/neuron-restrictive silencing factor (REST/NRSF) target genes. Proc. Natl. Acad. Sci. U S A 101, 10458–10463. 10.1073/pnas.0401827101 15240883 PMC478591

[B20] Champagne F. A. Curley J. P. (2009). Epigenetic mechanisms mediating the long-term effects of maternal care on development. Neurosci. Biobehav. Rev. 33, 593–600. 10.1016/j.neubiorev.2007.10.009 18430469

[B21] Chao M. V. Rajagopal R. Lee F. S. (2006). Neurotrophin signalling in health and disease. Clin. Sci. (Lond.) 110, 167–173. 10.1042/CS20050163 16411893

[B22] Chiu S. Woodbury-Fariña M. A. Shad M. U. Husni M. Copen J. Bureau Y.. (2014). The role of nutrient-based epigenetic changes in buffering against stress, aging and Alzheimer’s disease. Psychiatr. Clin. North Am. 37, 591–623. 10.1016/j.psc.2014.09.001 25455068

[B23] Chouliaras L. Rutten B. P. Kenis G. Peerbooms O. Visser P. J. Verhey F.. (2010). Epigenetic regulation in the pathophysiology of Alzheimer’s disease. Prog. Neurobiol. 90, 498–510. 10.1016/j.pneurobio.2010.01.002 20097254

[B24] Colman R. J. Beasley T. M. Kemnitz J. W. Johnson S. C. Weindruch R. Anderson R. M. (2014). Caloric restriction reduces age-related and all-cause mortality in rhesus monkeys. Nat. Commun. 5:3557. 10.1038/ncomms4557 24691430 PMC3988801

[B25] Coppedè F. (2012). Genetics and epigenetics of Parkinson’s disease. ScientificWorldJournal 2012:489830. 10.1100/2012/489830 22623900 PMC3353471

[B26] Coppedè F. (2014). The potential of epigenetic therapies in neurodegenerative diseases. Front. Genet. 5:220. 10.3389/fgene.2014.00220 25071843 PMC4094885

[B27] D’Alessandro R. Klajn A. Meldolesi J. (2009). Expression of dense-core vesicles and of their exocytosis are governed by the repressive transcription factor NRSF/REST. Ann. N Y Acad. Sci. 1152, 194–200. 10.1111/j.1749-6632.2008.03988.x 19161390

[B28] Day J. J. Sweatt J. D. (2010). DNA methylation and memory formation. Nat. Neurosci. 13, 1319–1323. 10.1038/nn.2666 20975755 PMC3130618

[B29] Day J. J. Sweatt J. D. (2011). Epigenetic mechanisms in cognition. Neuron 70, 813–829. 10.1016/j.neuron.2011.05.019 21658577 PMC3118503

[B30] Decottignies A. d’Adda di Fagagna F. (2011). Epigenetic alterations associated with cellular senescence: a barrier against tumorigenesis or a red carpet for cancer? Semin. Cancer Biol. 21, 360–366. 10.1016/j.semcancer.2011.09.003 21946622

[B31] Desplats P. Spencer B. Coffee E. Patel P. Michael S. Patrick C.. (2011). Alpha-synuclein sequesters Dnmt1 from the nucleus: a novel mechanism for epigenetic alterations in Lewy body diseases. J. Biol. Chem. 286, 9031–9037. 10.1074/jbc.C110.212589 21296890 PMC3059002

[B32] Draker R. Cheung P. (2009). Transcriptional and epigenetic functions of histone variant H2A.Z. Biochem. Cell Biol. 89, 19–25. 10.1139/O08-117 19234520

[B33] Dulac C. (2010). Brain function and chromatin plasticity. Nature 465, 728–735. 10.1038/nature09231 20535202 PMC3075582

[B34] Formisano L. Noh K. M. Miyawaki T. Mashiko T. Bennett M. V. Zukin R. S. (2007). Ischemic insults promote epigenetic reprogramming of mu opioid receptor expression in hippocampal neurons. Proc. Natl. Acad. Sci. U S A 104, 4170–4175. 10.1073/pnas.0611704104 17360495 PMC1820727

[B35] Fraga M. F. Ballestar E. Paz M. F. Ropero S. Setien F. Ballestar M. L.. (2005). Epigenetic differences arise during the lifetime of monozygotic twins. Proc. Natl. Acad. Sci. U S A 102, 10604–10609. 10.1073/pnas.0500398102 16009939 PMC1174919

[B36] Francis Y. I. Fà M. Ashraf H. Zhang H. Staniszewski A. Latchman D. S.. (2009). Dysregulation of histone acetylation in the APP/PS1 mouse model of Alzheimer’s disease. J. Alzheimers Dis. 18, 131–139. 10.3233/JAD-2009-1134 19625751 PMC8962655

[B37] Frisardi V. Panza F. Seripa D. Imbimbo B. P. Vendemiale G. Pilotto A.. (2010). Nutraceutical properties of Mediterranean diet and cognitive decline: possible underlying mechanisms. J. Alzheimers Dis. 22, 715–740. 10.3233/JAD-2010-100942 20858954

[B38] Fuso A. Cavallaro R. A. Zampelli A. D’Anselmi F. Piscopo P. Confaloni A.. (2007). γ-Secretase is differentially modulated by alterations of Homocysteine cycle in neuroblastoma and glioblastoma cells. J. Alzheimers Dis. 11, 275–290. 17851177 10.3233/jad-2007-11303

[B39] Fuso A. Nicolia V. Ricceri L. Cavallaro R. A. Isopi E. Mangia F.. (2012). S-adenosylmethionine reduces the progress of the Alzheimer-like features induced by B-vitamin deficiency in mice. Neurobiol. Aging 33, 1482.e1–1482.e16. 10.1016/j.neurobiolaging.2011.12.013 22221883

[B40] Goers J. Manning-Bog A. B. McCormack A. L. Millett I. S. Doniach S. Di Monte D. A.. (2003). Nuclear localization of α-synuclein and its interaction with histones. Biochemistry 42, 8465–8471. 10.1021/bi0341152 12859192

[B41] Goll M. G. Bestor T. H. (2005). Eukaryotic cytosine methyltransferases. Annu. Rev. Biochem. 74, 481–514. 10.1146/annurev.biochem.74.010904.153721 15952895

[B42] Greene A. E. Todorova M. T. Seyfried T. N. (2003). Perspectives on the metabolic management of epilepsy through dietary reduction of glucose and elevation of ketone bodies. J. Neurochem. 86, 529–537. 10.1046/j.1471-4159.2003.01862.x 12859666

[B43] Hardy J. (2006). A hundred years of Alzheimer’s disease research. Neuron 52, 3–13. 10.1016/j.neuron.2006.09.016 17015223

[B44] Harrison I. F. Dexter D. T. (2013). Epigenetic targeting of histone deacetylase: therapeutic potential in Parkinson’s disease? Pharmacol. Ther. 140, 34–52. 10.1016/j.pharmthera.2013.05.010 23711791

[B45] Heck N. Garwood J. Loeffler J. P. Larmet Y. Faissner A. (2004). Differential upregulation of extracellular matrix molecules associated with the appearance of granule cell dispersion and mossy fiber sprouting during epileptogenesis in a murine model of temporal lobe epilepsy. Neuroscience 129, 309–324. 10.1016/j.neuroscience.2004.06.078 15501589

[B46] Henikoff S. Furuyama T. Ahmad K. (2004). Histone variants, nucleosome assembly and epigenetic inheritance. Trends. Genet. 20, 320–326. 10.1016/j.tig.2004.05.004 15219397

[B47] Hernandez D. G. Nalls M. A. Gibbs J. R. Arepalli S. van der Brug M. Chong S.. (2011). Distinct DNA methylation changes highly correlated with chronological age in the human brain. Hum. Mol. Genet. 20, 1164–1172. 10.1093/hmg/ddq561 21216877 PMC3043665

[B48] Ho E. Clarke J. D. Dashwood R. H. (2009). Dietary sulforaphane, a histone deacetylase inhibitor for cancer prevention. J. Nutr. 139, 2393–2396. 10.3945/jn.109.113332 19812222 PMC2777483

[B49] Houser C. R. (1990). Granule cell dispersion in the dentate gyrus of humans with temporal lobe epilepsy. Brain Res. 535, 195–204. 10.1016/0006-8993(90)91601-c 1705855

[B50] International Parkinson’s Disease Genomics Consortium (IPDGC); Wellcome Trust Case Control Consortium 2 (WTCCC2). (2011). A two-stage meta-analysis identifies several New Loci for Parkinson’s disease. PLoS Genet. 7:e1002142. 10.1371/journal.pgen.1002142 21738488 PMC3128098

[B51] Jacinto F. V. Ballestar E. Esteller M. (2009). Impaired recruitment of the histone methyltransferase DOT1L contributes to the incomplete reactivation of tumor suppressor genes upon DNA demethylation. Oncogene 28, 4212–4224. 10.1038/onc.2009.267 19734945

[B52] Jarome T. J. Thomas J. S. Lubin F. D. (2014). The epigenetic basis of memory formation and storage. Prog. Mol. Biol. Transl. Sci. 128, 1–27. 10.1016/b978-0-12-800977-2.00001-2 25410539

[B53] Jeltsch A. (2006). On the enzymatic properties of Dnmt1: specificity, processivity, mechanism of linear diffusion and allosteric regulation of the enzyme. Epigenetics 1, 63–66. 10.4161/epi.1.2.2767 17965604

[B54] Jin C. Zang C. Wei G. Cui K. Peng W. Zhao K.. (2009). H3.3/H2A.Z double variant-containing nucleosomes mark ‘nucleosome-free regions’ of active promoters and other regulatory regions. Nat. Genet. 41, 941–947. 10.1038/ng.409 19633671 PMC3125718

[B55] Johnson R. Gamblin R. J. Ooi L. Bruce A. W. Donaldson I. J. Westhead D. R.. (2006). Identification of the REST regulon reveals extensive transposable element-mediated binding site duplication. Nucleic Acids Res. 34, 3862–3877. 10.1093/nar/gkl525 16899447 PMC1557810

[B56] Jowaed A. Schmitt I. Kaut O. Wüllner U. (2010). Methylation regulates alpha-synuclein expression and is decreased in Parkinson’s disease patients’ brains. J. Neurosci. 30, 6355–6359. 10.1523/JNEUROSCI.6119-09.2010 20445061 PMC6632710

[B57] Katada S. Imhof A. Sassone-Corsi P. (2012). Connecting threads: epigenetics and metabolism. Cell 148, 24–28. 10.1016/j.cell.2012.01.001 22265398

[B58] Kidd S. K. Schneider J. S. (2010). Protection of dopaminergic cells from MPP+-mediated toxicity by histone deacetylase inhibition. Brain Res. 1354, 172–178. 10.1016/j.brainres.2010.07.041 20654591 PMC2941155

[B59] Klose R. J. Bird A. P. (2006). Genomic DNA methylation: the mark and its mediators. Trends. Biochem. Sci. 31, 89–97. 10.1016/j.tibs.2005.12.008 16403636

[B60] Kobow K. Kaspi A. Harikrishnan K. N. Kiese K. Ziemman M. Khurana I.. (2013). Deep sequencing reveals increased DNA methylation in chronic rat epilepsy. Acta Neuropathol. 126, 741–756. 10.1007/s00401-013-1168-8 24005891 PMC3825532

[B61] Kolosova N. G. Shcheglova T. V. Sergeeva S. V. Loskutova L. V. (2006). Long-term antioxidant supplementation attenuates oxidative stress markers and cognitive deficits in senescent-accelerated OXYS rats. Neurobiol. Aging 27, 1289–1297. 10.1016/j.neurobiolaging.2005.07.022 16246464

[B62] Kontopoulos E. Parvin J. Feany M. (2006). Alpha-synuclein acts in the nucleus to inhibit histone acetylation and promote neurotoxicity. Hum. Mol. Genet. 15, 3012–3023. 10.1093/hmg/ddl243 16959795

[B63] Kroeger C. M. Klempel M. C. Bhutani S. Trepanowski J. F. Tangney C. C. Varady K. A. (2012). Improvement in coronary heart disease risk factors during an intermittent fasting/calorie restriction regimen: relationship to adipokine modulations. Nutr. Metab. (Lond) 9:98. 10.1186/1743-7075-9-98 23113919 PMC3514278

[B64] Kundakovic M. Gudsnuk K. Herbstman J. B. Tang D. Perera F. P. Champagne F. A. (2014). DNA methylation as a biomarker of early life adversity. Proc. Natl. Acad. Sci. U S A [Epub ahead of print]. 10.1073/pnas.1408355111 25385582 PMC4460453

[B65] LaPlant Q. Vialou V. Covington H. E. 3rd Dumitriu D. Feng J. Warren B. L.. (2010). Dnmt3a regulates emotional behavior and spine plasticity in the nucleus accumbens. Nat. Neurosci. 13, 1137–1143. 10.1038/nn.2619 20729844 PMC2928863

[B66] Lee C. Raffaghelio L. Brandhorst S. Safdie F. M. Bianchi G. Martin-Montalvo A.. (2012). Fasting cycles retard growth of tumors and sensitize a range of cancer cell types to chemotherapy. Sci. Transl. Med. 4:124ra27. 10.1126/scitranslmed.3003293 22323820 PMC3608686

[B67] Levenson J. M. Sweatt T. J. D. (2005). Epigenetic mechanisms in memory formation. Nat. Rev. Neurosci. 6, 108–118. 10.1038/nrn1604 15654323

[B68] Li B. Carey M. Workman J. L. (2007). The role of chromatin during transcription. Cell 128, 707–719. 10.1016/j.cell.2007.01.015 17320508

[B69] Lithner C. U. Lacor P. N. Zhao W. Q. Mustafiz T. Klein W. L. Sweatt J. D.. (2013). Disruption of neocortical histone H3 homeostasis by soluble Aβ: implications for Alzheimer’s disease. Neurobiol. Aging 34, 2081–2090. 10.1016/j.neurobiolaging.2012.12.028 23582659

[B70] Lu T. Aron L. Zullo J. Pan Y. Kim H. Chen Y.. (2014). REST and stress resistance in ageing and Alzheimer’s disease. Nature 507, 448–454. 10.1038/nature13163 24670762 PMC4110979

[B71] Maloney B. Sambamurti K. Zawia N. Lahiri D. K. (2012). Applying epigenetics to Alzheimer’s disease via the latent early-life associated regulation (LEARn) model. Curr. Alzheimer Res. 9, 589–599. 10.2174/156720512800617955 22300406

[B72] Marques J. F. Cappa S. F. Sartori G. (2011). Naming from definition, semantic relevance and feature type: the effects of aging and Alzheimer’s disease. Neuropsychology 25, 105–113. 10.1037/a0020417 20919761

[B73] Marsh E. B. Freeman J. M. Kossoff E. H. Vining E. P. Rubenstein J. E. Pyzik P. L.. (2006). The outcome of children with intractable seizures: a 3- to 6-year follow-up of 67 children who remained on the ketogenic diet less than one year. Epilepsia 47, 425–430. 10.1111/j.1528-1167.2006.00439.x 16499771

[B74] Mastroeni D. Grover A. Delvaux E. Whiteside C. Coleman P. D. Rogers J. (2011). Epigenetic mechanisms in Alzheimer’s disease. Neurobiol. Aging 32, 1161–1180. 10.1016/j.neurobiolaging.2010.08.017 21482442 PMC3115415

[B75] Migliore L. Coppedè F. (2009). Genetics, environmental factors and the emerging role of epigenetics in neurodegenerative diseases. Mutat. Res. 667, 82–97. 10.1016/j.mrfmmm.2008.10.011 19026668

[B76] Miller A. L. (2003). The methionine-homocysteine cycle and its effects on cognitive diseases. Altern. Med. Rev. 8, 7–19. 12611557

[B77] Morrison L. D. Smith D. D. Kish S. J. (1996). Brain S-adenosylmethionine levels are severely decreased in Alzheimer’s disease. J. Neurochem. 67, 1328–1331. 10.1046/j.1471-4159.1996.67031328.x 8752143

[B78] Müller H. de Toledo F. W. Resch K. L. (2001). Fasting followed by vegetarian diet in patients with rheumatoid arthritis: a systematic review. Scand. J. Rheumatol. 30, 1–10. 10.1080/030097401750065256 11252685

[B117] Nalls M. A. Bras J. Hernandez D. G. Keller M. F. Majounie E. Renton A. E.. (2014). `NeuroX, a fast and efficient genotyping platform for investigation of neurodegenerative diseases. Neurobiol. Aging [Epub ahead of print]. , pii:S0197-4580(14)00497-7. 10.1016/j.neurobiolaging.2014.07.028 25444595 PMC4317375

[B79] Newman J. C. Verdin E. (2014). Ketone bodies as signaling metabolites. Trends. Endocrinol. Metab. 25, 42–52. 10.1016/j.tem.2013.09.002 24140022 PMC4176946

[B80] Noh K. M. Hwang J. Y. Follenzi A. Athanasiadou R. Miyawaki T. Greally J. M.. (2012). Repressor element-1 silencing transcription factor (REST) dependent epigenetic remodeling is critical to ischemia-induced neuronal death. Proc. Natl. Acad. Sci. U S A 109, E962–E971. 10.1073/pnas.1121568109 22371606 PMC3341013

[B81] Nuytemans K. Theuns J. Cruts M. Van Broeckhoven C. (2010). Genetic etiology of Parkinson disease associated with mutations in the SNCA, PARK2, PINK1, PARK7 and LRRK2 genes: a mutation update. Hum. Mutat. 31, 763–780. 10.1002/humu.21277 20506312 PMC3056147

[B82] Outeiro T. F. Kontopoulos E. Altmann S. M. Kufarev A. I. Strathearn K. E. Amore A. M.. (2007). Sirtuin 2 inhibitors rescue alpha-synuclein-mediated toxicity in models of Parkinson’s disease. Science 317, 516–519. 10.1126/science.1143780 17588900

[B83] Palm K. Belluardo N. Metsis M. Timmusk T. (1998). Neuronal expression of zinc finger transcription factor REST/NRSF/XBR gene. J. Neurosci. 18, 1280–1296. 9454838 10.1523/JNEUROSCI.18-04-01280.1998 PMC6792720

[B84] Patel A. Pyzik P. L. Turner Z. Rubenstein J. E. Kossoff E. H. (2010). Longterm outcomes of children treated with the ketogenic diet in the past. Epilepsia 51, 1277–1282. 10.1111/j.1528-1167.2009.02488.x 20132287

[B85] Peleg S. Sananbenesi F. Zovoilis A. Burkhardt S. Bahari-Javan S. Agis-Balboa R. C.. (2010). Altered histone acetylation is associated with age-dependent memory impairment in mice. Science 328, 753–756. 10.1126/science.1186088 20448184

[B86] Pozzi D. Lignani G. Ferrea E. Contestabile A. Paonessa F. D’Alessandro R.. (2013). REST/NRSF-mediated intrinsic homeostasis protects neuronal networks from hyperexcitability. EMBO J. 32, 2994–3007. 10.1038/emboj.2013.231 24149584 PMC3831314

[B87] Prokhortchouk E. Defossez P. A. (2008). The cell biology of DNA methylation in mammals. Biochim. Biophys. Acta 1783, 2167–2173. 10.1016/j.bbamcr.2008.07.015 18706939

[B88] Qing H. He G. Ly P. T. T. Fox C. J. Staufenbiel M. Cai F.. (2008). Valproic acid inhibits Abeta production, neuritic plaque forma-tion and behavioral deficits in Alzheimer’s disease mouse models. J. Exp. Med. 205, 2781–2788. 10.1084/jem.20081588 18955571 PMC2585842

[B89] Renthal W. Maze I. Krishnan V. Covington H. E. 3rd Xiao G. Kuma R. A.. (2007). Histone deacetylase 5 epigenetically controls behavioral adaptations to chronic emotional stimuli. Neuron 56, 517–529. 10.1016/j.neuron.2007.09.032 17988634

[B90] Renthal W. Nestler E. J. (2008). Epigenetic mechanisms in drug addiction. Trends Mol. Med. 14, 341–350. 10.1016/j.molmed.2008.06.004 18635399 PMC2753378

[B91] Roopra A. Dingledine R. Hsieh J. (2012). Epigenetics and epilepsy. Epilepsia 53(Suppl. 9), 2–10. 10.1111/epi.12030 23216574 PMC3531878

[B92] Rothbart S. B. Strahl B. D. (2014). Interpreting the language of histone and DNA modifications. Biochim. Biophys. Acta 1839, 627–643. 10.1016/j.bbagrm.2014.03.001 24631868 PMC4099259

[B93] Sassone-Corsi P. (2013). Physiology. When metabolism and epigenetics converge.. Science 339, 148–150. 10.1126/science.1233423 23307727

[B94] Scarmeas N. Stern Y. Mayeux R. Manly J. J. Schupf N. Luchsinger J. A. (2009). Mediterranean diet and mild cognitive impairment. Arch. Neurol. 66, 216–225. 10.1001/archneurol.2008.536 19204158 PMC2653223

[B95] Sezgin Z. Dincer Y. (2014). Alzheimer’s disease and epigenetic diet. Neurochem. Int. 78, 105–116. 10.1016/j.neuint.2014.09.012 25290336

[B96] Shahbazian M. D. Grunstein M. (2007). Functions of site-specific histone acetylation and deacetylation. Annu. Rev. Biochem. 76, 75–100. 10.1146/annurev.biochem.76.052705.162114 17362198

[B97] Shimazu T. Hirschey M. D. Newman J. He W. Shirakawa K. Le Moan N.. (2013). Suppression of oxidative stress by β-hydroxybutyrate, an endogenous histone deacetylase inhibitor. Science 339, 211–224. 10.1126/science.1227166 23223453 PMC3735349

[B116] Shu L. Cheung K. L. Khor T. Chen C. Kong A. N. (2010). Phytochemicals: cancer chemoprevention and suppression of tumor onset and metastasis. Cancer Metastasis Rev. 29, 483–502. 10.1007/s10555-010-9239-y 20798979

[B98] Shyh-Chang N. Locasale J. W. Lyssiotis C. A. Zheng Y. Teo R. Y. Ratanasirintrawoot S.. (2013). Influence of threonine metabolism on S-adenosylmethionine and histone methylation. Science 339, 222–226. 10.1126/science.1226603 23118012 PMC3652341

[B99] Stafstrom C. E. Zupec-Kania B. Rho J. M. (2008). Epilepsia. Ketogenic diet and treatments. Introduction/perspectives. Epilepsia 49(Suppl. 8), 1–2. 10.1111/j.1528-1167.2008.01820.x 19049573

[B100] Strahl B. D. Allis C. D. (2000). The language of covalent histone modification. Nature 403, 41–45. 10.1038/47412 10638745

[B101] Su Y. Ryder J. Li B. Wu X. Fox N. Solenberg P.. (2004). Lithium, a common drug for bipolar disorder treatment, regulates amyloid-precursor protein processing. Biochemistry 43, 6899–6908. 10.1021/bi035627j 15170327

[B102] Sweatt J. D. (2013). The emerging field of neuroepigenetics. Neuron 80, 624–632. 10.1016/j.neuron.2013.10.023 24183015 PMC3878295

[B103] Thomas B. Beal M. F. (2011). Molecular insights into Parkinson’s disease. F1000 Med. Rep. 3:7. 10.3410/M3-7 21655332 PMC3096887

[B104] Tohgi H. Utsugisawa K. Nagane Y. Yoshimura M. Genda Y. Ukitsu M. (1999). Reduction with age in methylcytosine in the promoter region −224 approximately −101 of the amyloid precursor protein gene in autopsy human cortex. Brain Res. Mol. Brain Res. 70, 288–292. 10.1016/s0169-328x(99)00163-1 10407177

[B105] Tsankova N. Renthal W. Kumar A. Nestler E. (2007). Epigenetic regulation in psychiatric disorders. Nat. Rev. Neurosci. 8, 355–367. 10.1038/nrn2132 17453016

[B106] Varady K. A. Bhutani S. Klempel M. C. Kroeger C. M. Trepanowski J. F. Haus J. M.. (2013). Alternate day fasting for weight loss in normal weight and overweight subjects: a randomized controlled trial. Nutr. J. 12:146. 10.1186/1475-2891-12-146 24215592 PMC3833266

[B107] Waddington C. H. (1942). The epigenotype. Endeavour 1, 18–20.

[B108] Waddington C. H. (1968). Towards a Theoretical Biology. Edinburgh, Scotland: Edinburgh University Press.

[B109] Wang J. Yu J. T. Tan M. S. Jiang T. Tan L. (2013). Epigenetic mechanisms in Alzheimer’s disease: implications for pathogenesis and therapy. Ageing Res. Rev. 12, 1024–1041. 10.1016/j.arr.2013.05.003 23688931

[B110] Wu J. Basha M. R. Brock B. Cox D. P. Cardozo-Pelaez F. McPherson C. A.. (2008a). Alzheimer’s disease (AD)-like pathology in aged monkeys after infantile exposure to environmental metal lead (Pb): evidence for a developmental origin and environmental link for AD. J. Neurosci. 28, 3–9. 10.1523/JNEUROSCI.4405-07.2008 18171917 PMC2486412

[B111] Wu X. Chen P. S. Dallas S. Wilson B. Block M. L. Wang C. C.. (2008b). Histone deacetylase inhibitors up-regulate astrocyte GDNF and BDNF gene transcription and protect dopaminergic neurons. Int. J. Neuropsychopharmacol. 11, 1123–1134. 10.1017/S1461145708009024 18611290 PMC2579941

[B112] Xu Z. Li H. Jin P. (2012). Epigenetics-based therapeutics for neurodegenerative disorders. Curr. Transl. Geriatr. Exp. Gerontol. Rep. 1, 229–236. 10.1007/s13670-012-0027-0 23526405 PMC3601938

[B113] Yuen A. W. Sander J. W. (2014). Rationale for using intermittent calorie restriction as a dietary treatment for drug resistant epilepsy. Epilepsy Behav. 33, 110–114. 10.1016/j.yebeh.2014.02.026 24657501

[B114] Zetzsche T. Rujescu D. Hardy J. Hampel H. (2010). Advances and perspectives from genetic research: development of biological markers in Alzheimer’s disease. Expert Rev. Mol. Diagn. 10, 667–690. 10.1586/erm.10.48 20629514

[B115] Zovkic I. B. Guzman-Karlsson M. C. Sweatt J. D. (2013). Epigenetic regulation of memory formation and maintenance. Learn. Mem. 20, 61–74. 10.1101/lm.026575.112 23322554 PMC3549063

